# Adaptation to an amoeba host drives selection of virulence-associated traits in *Vibrio cholerae*

**DOI:** 10.1038/s41396-021-01134-2

**Published:** 2021-10-15

**Authors:** M. Mozammel Hoque, Parisa Noorian, Gustavo Espinoza-Vergara, Pradeep Manuneedhi Cholan, Mikael Kim, Md Hafizur Rahman, Maurizio Labbate, Scott A. Rice, Mathieu Pernice, Stefan H. Oehlers, Diane McDougald

**Affiliations:** 1 The iThree Institute, University of Technology Sydney, Sydney, NSW Australia; 2 Tuberculosis Research Program at the Centenary Institute, The University of Sydney, Camperdown, NSW Australia; 3 Faculty of Medicine and Health & Marie Bashir Institute, The University of Sydney, Camperdown, NSW Australia; 4 Climate Change Cluster, University of Technology Sydney, Sydney, NSW Australia; 5 School of Life Sciences, Faculty of Science, University of Technology Sydney, Sydney, NSW Australia; 6 Singapore Centre for Environmental Life Sciences Engineering, Nanyang Technological University, Singapore, Singapore

**Keywords:** Water microbiology, Bacterial genetics

## Abstract

Predation by heterotrophic protists drives the emergence of adaptive traits in bacteria, and often these traits lead to altered interactions with hosts and persistence in the environment. Here we studied adaptation of the cholera pathogen, *Vibrio cholerae* during long-term co-incubation with the protist host, *Acanthamoeba castellanii*. We determined phenotypic and genotypic changes associated with long-term intra-amoebal host adaptation and how this impacts pathogen survival and fitness. We showed that adaptation to the amoeba host leads to temporal changes in multiple phenotypic traits in *V. cholerae* that facilitate increased survival and competitive fitness in amoeba. Genome sequencing and mutational analysis revealed that these altered lifestyles were linked to non-synonymous mutations in conserved regions of the flagellar transcriptional regulator, *flrA*. Additionally, the mutations resulted in enhanced colonisation in zebrafish, establishing a link between adaptation of *V. cholerae* to amoeba predation and enhanced environmental persistence. Our results show that pressure imposed by amoeba on *V. cholerae* selects for *flrA* mutations that serves as a key driver for adaptation. Importantly, this study provides evidence that adaptive traits that evolve in pathogens in response to environmental predatory pressure impact the colonisation of eukaryotic organisms by these pathogens.

## Introduction

Predation by heterotrophic protists and bacteriophages represent a major driving force shaping bacterial population structure and composition [1, 2]. In response to predation pressure, bacteria have developed sophisticated mechanisms and adaptive traits which enhance their survival and persistence in the environment [3, 4]. Antipredator strategies are hypothesised to have evolved from predation pressure and play crucial roles in predation resistance and virulence, which supports the ‘coincidental evolution’ hypothesis [5–8]. The hypothesis states that, virulence arises as an accidental consequence of antipredator mechanisms in distinct niches rather than for virulence per se. In contrast, predation driven attenuation of virulence has also been observed and this results in increased commensalism between bacteria and hosts/predators [9–11]. Such commensal relationships enhance pathogen persistence and transmission in the environment.

Patho-adaptations to protist hosts are now widely recognised to function as training grounds for bacterial pathogens including *Vibrio cholerae*, the waterborne bacterium that causes the acute diarrhoeal disease, cholera [12]. Cholera follows a distinct pattern with recurring episodes of seasonal epidemics suggesting potential involvement of environmental factors in the survival of the organism in the environment [13], including interactions with heterotrophic protists [14]. It has been reported that the free-living amoeba, *Acanthamoeba castellanii*, and the ciliate, *Tetrahymena pyriformis*, serve as environmental hosts for *V. cholerae* [15, 16]. In fact, *T. pyriformis* and other ciliates expel a hyper-infectious form of *V. cholerae* packaged in released food vacuoles [5], suggesting that protists play an important role in the infection cycle of *V. cholerae* by increasing environmental resistance and persistence. A growing number of reports have described the roles of different phenotypic traits and their mechanisms of grazing resistance in *V. cholerae*. For example, changes in biofilm formation, secondary metabolite production (e.g. pyomelanin), extracellular proteases (e.g. HapA, PrtV) and quorum-sensing mediated production of anti-protozoal factors are involved in grazing resistance and association with diverse protist hosts [17–20]. Swimming motility controls the frequency of contact depending on the type of protist hosts. Increased swimming speed enhances grazing avoidance when encountering some flagellated protists [21], while enhancing the frequency of contact with raptorial feeding protists [22, 23]. Grazing resistance of biofilms largely depends on the type of protozoan grazers. Surface-feeding protozoa, such as amoeba preferentially feed on biofilm cells whereas suspension-feeding protozoa (i.e. ciliates and flagellates) feed mainly on planktonic cells [24]. Anti-protozoal activities of *V. cholerae* biofilms have been shown to provide grazing resistance to different protozoan hosts [20]. Extracellular proteases have also been shown to confer grazing resistance to flagellated and ciliated protozoa [17]. Other secreted factors, such as the hemolysin also play important roles in the intracellular survival of *V. cholerae* in protozoa [19]. However, many aspects of the interactions between protozoa and *V. cholerae* are yet to be explored, in particular the selective pressure exerted by long-term exposure to predation and the emergence of adaptive traits which allow the pathogen to persist in the environment.

This study aimed to identify adaptive traits that arise as a result of long-term co-adaptation of *V. cholerae* with *A. castellanii*. Changes in genotypic and phenotypic characteristics of *V. cholerae* in the presence and absence of amoeba for 90 days were investigated in populations as well as in individual isolates. The late-stage amoeba-adapted isolates showed increased survival and competitive fitness in amoeba. There was a decrease in motility, biofilm formation and haemolytic activity and an increase in protease activity in amoeba-adapted isolates compared to non-adapted isolates. We found that the altered phenotypic behaviours and improved fitness in late-stage amoeba-adapted *V. cholerae* was associated with mutations in the flagellar transcriptional master regulator, *flrA* (VC2137). Finally, we show that mutations in *flrA* increase the competitive fitness and colonisation potential of *V. cholerae* in zebrafish. Taken together, these results show that adaptation of *V. cholerae* in a natural host can drive the evolution of host–pathogen interactions.

## Materials and methods

### Organisms and growth conditions

*V. cholerae* O1 El Tor strain A1552 and its derivatives were routinely grown on Luria Bertani (LB) agar plates or in liquid LB medium with shaking. *A. castellanii* was routinely maintained axenically in peptone yeast glucose (PYG) medium supplemented with salts (ATCC medium 712) at room temperature in 25 cm^2^ tissue culture flasks with ventilated caps (Sarstedt Inc., Nümbrecht, Germany). *A. castellanii* was passaged 3 days prior to harvesting for experiments and enumerated microscopically using a hemocytometer. The long-term co-incubation was performed in (2M) marine minimal medium (1 M MOPS, pH 8.2; 132 mM K_2_HPO_4_; 952 mM NH_4_Cl; 0.4 M Tricine and 1 mM FeSO_4._7H_2_O, pH 7.8 in artificial seawater) supplemented with 0.08% glucose [25]. The artificial seawater, or nine salts solution (0.5 X NSS) was composed of 17.6 g NaCl, 1.47 g Na_2_SO_4_, 0.08 g NaHCO_3_, 0.25 g KCl, 0.04 g KBr, 1.87 g MgCl_2_•6H_2_O, 0.45 g CaCl_2_•2H_2_O, 0.01 g SrCl_2_•6H_2_O and 0.01 g H_3_BO_3_ in one liter of distilled water [26]. All organisms, oligonucleotide primers and plasmids used in this study are listed in Supplementary Table 1.

### Experimental co-adaptation of *V. cholerae* with *A. castellanii*

Three biological replicates of *V. cholerae* A1552 were grown overnight and washed with 2M medium [25], adjusted to 10^9^ cells ml^−1^ (OD_600_ = 1.0) and 100 µL of diluted cells (~10^7^ cells ml^−1^) were used to inoculate three independent cultures (P1, P2 and P3) of *A. castellanii* (1 × 10^5^ cells ml^−1^) in 1 mL of 2M medium in 24-well plates. The plates were incubated at room temperature with shaking at 60 rpm. Every three days, the amoeba in three replicate wells were lysed with 1% Triton X-100 and the released *V. cholerae* passaged to a fresh plate of amoeba. One half of the *V. cholerae* cells were placed at −80 °C for selection of individual isolates while the remainder was used for isolation of population genomic DNA for sequencing. In parallel as controls, three biological replicates of *V. cholerae* maintained in 2M medium in triplicate in the absence of amoeba were sub-cultured every three days in fresh 2 M medium after treating with 1% Triton X-100. The *V. cholerae* grown in presence or absence of *A. castellanii* were defined as adapted and non-adapted populations, respectively. The individual clones derived from the populations were defined as adapted and non-adapted isolates, respectively.

### Intracellular survival assays

*A. castellanii* was seeded at a concentration of 1 × 10^5^ cells ml^−1^ in 2M medium into 24-well plates 1 h prior to the addition of bacterial cells to allow them to adhere. Overnight amoeba-adapted and non-adapted isolates of *V. cholerae* were washed and diluted to OD_600_ 1.0 in 2M medium. Diluted cells (10^7^ cells) were used to inoculate triplicate wells of previously seeded *A. castellanii* at a final amoeba-bacteria ratio of 1:100. The plates were incubated statically at room temperature and intracellular bacteria were recovered at different time points by lysis with 1% Triton-X. Thirty minutes before lysis, 300 µg ml^−1^ of gentamicin (Sigma-Aldrich, United States) was added to kill extracellular bacteria followed by washing with 2M media to remove antibiotics. Intracellular bacteria were collected and resuspended in 2M medium before plating for enumeration. The percentage of intracellular survival was calculated for each of the isolates using the formula number of surviving bacteria at 4 h/number of surviving bacteria at 2 h.

### Competition assays

In vitro and in vivo competition assays were conducted by mixing a ∆*lacZ* strain of A1552 with the amoeba-adapted isolates at a ratio of 1:1 (v/v). For in vitro competition assays, 100 µL of the mixed bacterial cells (~10^7^ CFU ml^−1^) were inoculated into LB media and grown for 24 h. Serial dilutions of the cultures were plated on LB agar plates supplemented with 50 µg ml^−1^ X-gal (5-bromo-4-chloro-3-indolyl-D-galactopyranoside) which allowed enumeration of β-galactosidase-positive and negative *V. cholerae* colonies. For competition assays in amoeba, 100 µL (~10^7^ CFU ml^−1^) of the mixed bacterial cells were incubated with amoeba (~10^5^ cells ml^−1^) to a final amoeba-bacteria ratio of 1:100 for 24 h. Amoeba cells were lysed and intracellular bacteria were recovered as previously described. The competition index (CI) for each of the isolates were calculated by dividing the output ratio (adapted/∆*lacZ*) after incubation corrected by the input ratio.

### Motility assays

Swimming motility assays were performed on LB plates containing 0.3% (w/v) agar. An overnight colony of the *V. cholerae* isolates was inoculated by stab and the plates were incubated at 30 °C for 24 h and imaged (Bio-Rad, USA). The diameters of the migration zone were measured using ImageJ.

### Quantification of biofilm biomass

Biofilms were formed in microtiter plates and biomass was quantified by the crystal violet (CV) staining assay as described previously [27]. Briefly, *V. cholerae* clones were grown in LB to an OD_600_ of 0.6 (~10^6^ cells ml^−1^). The normalised suspensions were diluted 100-fold and inoculated into fresh LB and grown for 36 h at room temperature. The aqueous phase was removed and the attached biomass washed with 50% NSS. The CV solution (0.1%) was added and incubated for 10 min at room temperature. The plate was washed to remove unbound CV and biofilm-associated CV was solubilized with 95% ethanol, and the OD_600_ was measured using a micro-plate reader (Tecan Spark, Switzerland). Values were corrected by deducting the baseline defined as LB medium. The experiment was performed in triplicate and run in three independent experiments.

### Protease assay

Protease activity was determined by the azocasein assay as described previously [28]. Briefly, *V. cholerae* clones were grown overnight in LB at 37 °C with shaking at 200 rpm and adjusted to 10^9^ cells ml^−1^ (OD_600_ = 1.0). The suspension was filtered and 100 µL of cell-free supernatant was mixed with 900 µL of azocasein solution (1% in 0.1 M Tris pH 8.0) for 4 h at 37 °C and the reaction stopped with the addition of equal volumes of 10 % trichloroacetic acid. Precipitated, undegraded protein was removed by centrifugation, the supernatant collected, and the absorbance determined by spectrophotometry (450 nm). The enzymatic activity unit was determined by the following formula U = Abs at 450 nm/conc. of substrate (g)/time (hour) and the activity unit standardised by OD_600_ of the culture prior to centrifugation.

### Hemolysin assay

Hemolysin assays were performed using previously described methods with some modifications [29]. *V. cholerae* isolates grown in LB were centrifuged, washed and cell numbers adjusted to 10^9^ cells ml^−1^ (OD_600_ = 1.0) in PBS (pH 7.4). Equal volumes of cells were mixed with 1% sheep erythrocyte suspensions and incubated at 37 °C for 2 h followed by incubation at 4 °C for 1 h. Unlysed RBCs were removed by centrifugation and haemolytic activity measured by spectrophotometry at 540 nm. Percentage of haemolysis was calculated using the formula (sample absorbance − blank absorbance)/(control absorbance − blank absorbance) × 100. As a positive control cells were lysed with 1% Triton X-100.

### Sequencing and genomic analysis

Genomic DNA from three replicates (P1, P2, P3) of amoeba-adapted and non-adapted *V. cholerae* populations from days 3, 45 and 90 were subjected to genome sequencing and analysed for the presence of mutations (Supplementary Data 1 and Supplementary Data 2). In addition to whole population sequencing, 18 individual amoeba-adapted and non-adapted isolates were randomly selected (three from each population) for sequencing and analysis (Supplementary Data 3 and Supplementary Data 4).

Genomic DNA was extracted using the QIAamp DNA mini kit (Qiagen) according to manufacturer’s instructions. DNA concentration was measured using the Qubit dsDNA high sensitivity assay kit (Life Technologies). DNA libraries were prepared using TruSeq DNA sample preparation kit (Illumina, San Diego, CA, USA), and sequenced on a MiSeq (Illumina, USA) to generate 2 × 150 bp paired-end reads at the Singapore Centre for Environmental Life Sciences Engineering, Nanyang Technological University, Singapore. Sequenced reads were trimmed to remove adapter contamination and low-quality bases (≤Q20) using TrimGalore and were checked using FastQC before analysis (https://www.bioinformatics.babraham.ac.uk/projects/trim_galore/. Accessed Nov 2019) [30].

Filtered reads were mapped to the *V. cholerae* O1 El Tor strain A1552 (NCBI GenBank accession no CP025936 and CP025937 for chromosome I and II respectively) and genetic variants including single nucleotide polymorphisms (SNPs) and insertions and deletions (INDELS) were called along with the respective genotype information of samples using the polymorphism mode of breseq pipeline with a polymorphism frequency cut off of 0.1 [31]. Mutation calls were manually curated to remove false positives and only the mutations that arose during the co-incubation period were considered. Point mutations including synonymous single nucleotide polymorphisms (sSNPs), non-synonymous SNPs (nsSNPs), intergenic mutations and INDELs (<50 bp) were identified. The numbers and types of genetic alterations identified in adapted and non-adapted populations and single isolates are depicted in Supplementary Fig. 1A, B. Short paired-end reads from the single isolates were assembled into contigs using SPAdes 3.11.1 [32]. The assembled genomes were used to design primers to detect SNPs by ARMS-PCR and to analyse the mutations by alignment using BLASTn. Multiple sequence alignment was performed on the T-coffee server and annotated using the Expasy Box shade tool [33]. All the populations and single isolates sequences were deposited under NCBI bio project accession number PRJNA685017.

### Structural modelling of the FlrA AAA+ domain

Comparative modelling of the AAA+ domain of FlrA was generated using MolStar on the Protein Data Bank web server [34]. The crystal structure of the AAA+ domain of FleQ from *Pseudomonas aeruginosa* (Protein Data Bank code 5EXP) was used as the modelling template [35]. The amino acid residues were manually denoted according to the nsSNPs in the AAA+ domain.

### Amplification refractory mutation system (ARMS)-PCR

ARMS-PCR were used to detect the single point mutations in *flrA*. The online primer1 tool (http://primer1.soton.ac.uk/primer1.html) was used to design allele specific primers. Briefly, a mismatch at the −2 position of the 3′ end of the primer was introduced following wild type or mutant alleles using principles establish for ARMS-PCR [36]. In addition, a common primer located at the 5′ and 3′ terminus of *flrA* were designed. These four primer sets were used in a single PCR reaction to detect wild type and mutant alleles. The presence of an allele was confirmed by size differences of allele specific amplicons in agarose gel electrophoresis. Primers targeting the A213V (C638T) and V261G (T782G) mutations of *flrA* are listed in Supplementary Table 1.

### Generation and complementation of *flrA* mutant

The *V. cholerae* ∆*flrA* mutant was constructed by splicing overlap extension PCR (SOE PCR) and natural transformation [37]. Briefly, primers were used to amplify two flanking regions of the *flrA* gene (VC2137) and fused with the chloramphenicol acetyltransferase (*cat*) gene amplified from pKD3 using SOE PCR. The construct was transformed into *V. cholerae* using chitin-mediated natural transformation [38]. The transformants were selected on LB agar plates supplemented with 5 µg ml^−1^ of chloramphenicol. The *cat* gene was removed from the resultant transformants using the TransFLP method to get an in-frame deletion mutant [39]. The absence of the gene was confirmed by PCR.

For complementation, *flrA* from wild type A1552 as well as the mutants (A213V and V261G) from amoeba-adapted isolates were amplified by PCR and cloned into pBAD24 using Gibson assembly [40]. The recombinant plasmids were transformed into *E. coli* using chemical transformation and recombinant transformants selected on LB agar plates containing 100 µg ml^−1^ of ampicillin. Recombinant plasmids were extracted from *E. coli* using a GeneJET plasmid miniprep kit (Thermo Fisher Scientific). The recombinant plasmids were transformed into the *V. cholerae* ∆*flrA* mutant using chitin-mediated transformation. The transformants were selected on LB agar plates supplemented with 100 µg ml^−1^ of ampicillin. Sequences of all genes were verified using PCR for the WT copy and ARMS-PCR for the mutated copy with primers in Supplementary Table 1.

### Scanning electron microscopy

Bacterial cultures were fixed with 2 % glutaraldehyde in PBS and then stored in PBS at 4 °C until processing. Twenty microliters of undiluted bacteria samples were vacuum filtered onto polycarbonate filters (diameter 47 mm, pore size 0.4 μm). The preparation was subjected to a dehydration series with ethanol and milli-Q water (35, 50, 75, and 100% ethanol for 10 min each) followed by hexamethyldisilazane (HMDS) drying (50 and 100% for 10 min each). Filters were mounted onto SEM mounts with carbon tape and air dried for 24 h before sputter coating with 10 nm of gold/palladium. Samples were imaged via an SEM (Zeiss supra 55).

### Fluorescent tagging

Green fluorescent protein (GFP) and red fluorescent protein (DsRedExpress)-tagged strains were generated using mini-Tn7 delivery plasmids and the helper plasmid pUX-BF13 containing the Tn7 transposase gene as described previously [41]. For zebrafish experiments, the test strains were tagged with GFP and the isogenic Δ*lacZ* strain was tagged with RFP. Swapping of the fluorescent markers was previously tested in isogenic strains and did not alter other phenotypic traits [5]. Plasmids were transformed using chitin-mediated natural transformation into bacterial strains and transconjugants were selected on LB agar medium containing 50 µg ml^−1^ gentamicin at 30 °C. Fluorescent tagged strains were verified by PCR analysis and fluorescence microscopy.

### Adult zebrafish infection and histology

Adult zebrafish (*Danio rerio*) infection experiments were carried out with ethical approval from the Sydney Local Health District Animal Welfare Committee approval 19–031. Zebrafish were raised to 6–12 months of age in a recirculating aquarium system. Animals were transferred to a 28 °C incubator with a 14:10 h light:dark cycle in 1 L beakers for overnight acclimatisation. Zebrafish were exposed to 5 × 10^9^ CFU of *V. cholerae* test and *lacZ* negative strains for 6 h in 200 ml of aquarium system water yielding a final concentration of 2.5 × 10^7^ CFU ml^−1^ [42]. Zebrafish were washed in a clean water system and housed until 24 h post infection. Zebrafish were euthanised by tricaine (MS-222, Sigma) overdose and intestines were dissected. Dissected intestines were homogenised in PBS using a bead beater at 4 °C and homogenates plated on LB agar containing rifampicin (50 µg ml^−1^) and X-gal (80 µg ml^−1^) for enumeration. The CI was calculated by dividing the output ratio (test strain/∆*lacZ*) after infection corrected by the input ratio.

For the histology, dissected intestines were fixed in 10 % neutral buffered formalin overnight at 4 °C, rinsed in PBS, and then incubated in 30% (w/v) sucrose, 50:50 30 % sucrose:OCT (4583, Tissue-Tek), and OCT for ~4 h each [43]. Tissue was frozen in OCT and cryosectioned in 20 μm sections. Slides were rinsed in PBS, coverslips were mounted with DAPI fluoromount G (ProSciTech), and imaging was carried out on a Leica DM6000B microscope [43]. Fluorescent bacteria were quantified by fluorescent pixel count of sections in ImageJ [44]. Data are pooled from two animals per group.

### Statistical analysis

GraphPad Prism software version 9 La Jolla California USA, (www.graphpad.com) was used for statistical analysis. Two-tailed student’s *t* tests (non-parametric Mann–Whitney tests) were used to compare means between WT and mutant bacteria. Statistical analysis for experiments with multiple samples were performed using either two-way ANOVA and Sidak’s multiple comparisons test or one-way ANOVA and Kruskal–Wallis test. Principal component analysis (PCA) was conducted in rstudio using the ‘ggfortify’ package (https://cran.r-project.org/web/packages/ggfortify/index.html).

## Results

We tested the effects of long-term co-adaptation of *V. cholerae* to the amoeba predator, *A. castellanii*. It has been suggested that long-term predation can result in genotypic and phenotypic changes in bacterial prey and that these changes can affect environmental persistence and colonisation of host organisms. Here, *V. cholerae* was co-incubated with amoeba for 90 days in triplicate and intracellular bacteria were collected from amoeba in each replicate every 3 days. Samples of *V. cholerae* incubated in 2M medium only were also taken every 3 days as controls. To identify phenotypic changes, we first obtained individual isolates from both the co-incubation and control samples taken on days 3, 45 and 90. To identify the underlying mechanisms driving these phenotypic changes, sequencing of both the populations and the individual isolates, from both the co-incubation and control samples obtained at the 3 times points were performed.

### Increased intracellular survival and competitive fitness of amoeba-adapted isolates

Three biological replicates (P1, P2, and P3) of *V. cholerae* were grown in the presence and absence of amoeba for 90 days in marine minimal (2M) medium. In addition, individual *V. cholerae* isolates were collected every 3 days; adapted isolates were recovered from the co-cultures while non-adapted isolates were collected from 2M without amoeba. In total, 90 adapted and 90 non-adapted isolates were recovered, 30 from each treatment on days 3, 45 and 90. In order to assess differences in bacterial fitness, the intra-amoebal survival of the adapted isolates were assessed and compared to the non-adapted isolates using a gentamicin protection assay [45]. The gentamicin protection assay tests for the internalisation of bacterial cells as the antibiotic cannot penetrate eukaryotic cells, thus allowing for protection of intracellular bacteria against the antibiotic.

**Fig. 1:**
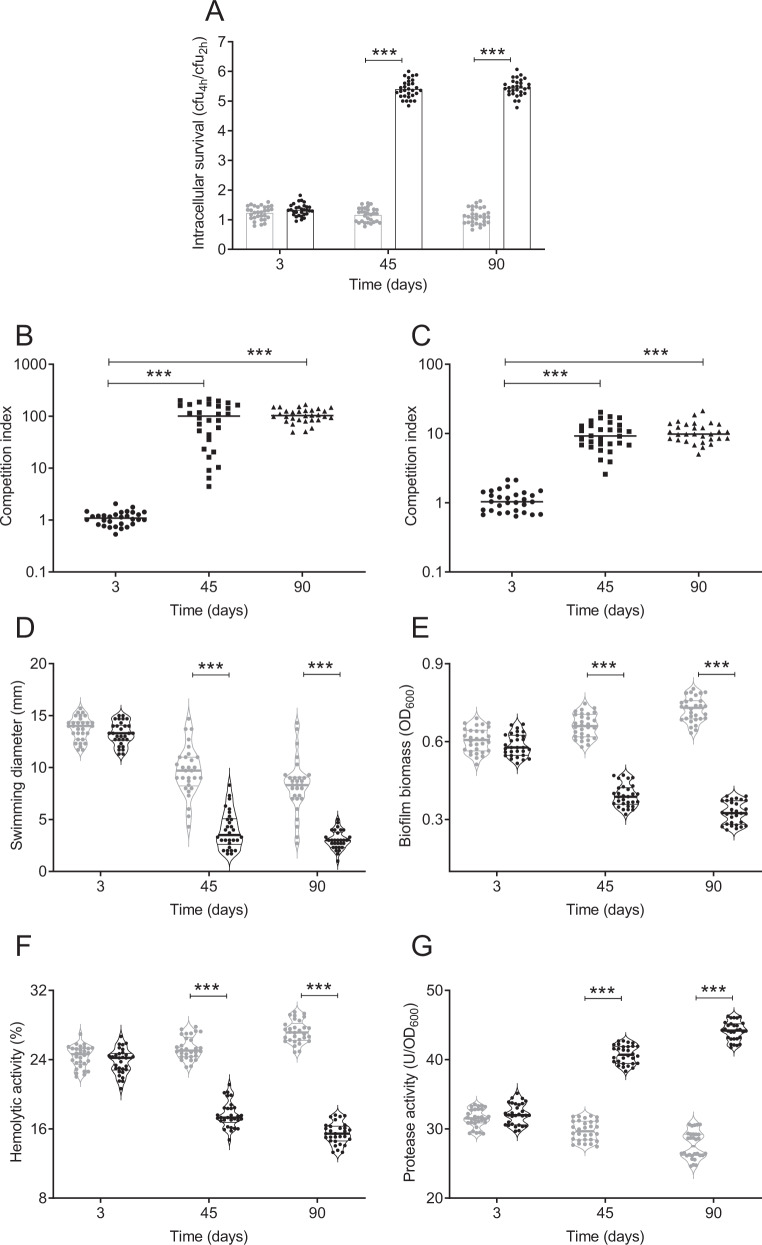
Intracellular survival, competitive fitness and virulence phenotypes of adapted and non-adapted isolates. **A** Intracellular survival in *A. castellanii* of adapted (black) and non-adapted (grey) isolates calculated by dividing the CFU at 4 h by the CFU at 2 h of the assay. **B** Intracellular competitive fitness of adapted isolates in *A. castellanii*. **C** In vitro competition assay of adapted isolates grown in LB for 24 h. Competition index (CI) in amoeba (**B**) and in LB medium (**C**) was calculated by dividing the output ratio (adapted/*∆lacZ*) after incubation, corrected by the input ratio. Statistical analysis was performed to determine the significance of the CI of days 45 and 90 isolates compared to day 3 isolates. **D** Swimming motility of the adapted (black) and non-adapted (grey) isolates expressed as diameter (mm) of the zone travelled from point of inoculation by bacteria grown overnight on semi-solid LB agar plates. **E** Biofilm biomass of adapted (black) and non-adapted (grey) isolates as quantified by crystal violet staining. **F** Haemolytic activity of adapted (black) and non-adapted (grey) isolates expressed as percent haemolysis of 1% sheep erythrocyte suspension. **G** Protease activity of cell-free supernatants of adapted (black) and non-adapted (grey) isolates measured by azocasein hydrolysis. Data are obtained from 30 individual adapted and non-adapted isolates from 3 time points (days 3, 45 and 90). Data are shown as the median. Statistical analyses for (**A**, **D**, **E**, **F**, **G**) were performed using two-way ANOVA and Sidak’s multiple comparisons test. For (**B**, **C**) statistical analyses were performed using one-way ANOVA and Kruskal–Wallis test. Statistical significance is indicated by ^∗∗∗^*p* < 0.001.

The intracellular survival of day 3 adapted and non-adapted isolates was not significantly different, while 45 and 90 day adapted isolates showed a fivefold increased survival compared to the non-adapted isolates (Fig. 1A). To further confirm the increased intracellular survival of the adapted isolates, an intracellular competition experiment was performed using a ∆*lacZ* isogenic strain. We calculated the competitive index (CI) from the competition experiment to assess the competitive fitness of the isolates in amoeba as the CI measures the fitness of bacteria in the condition tested [46]. After 24 h of incubation, day 3 adapted isolates showed a CI of ~1.0, while the CI of 45 and 90 day adapted isolates were 100.8 and 102.8, respectively, indicating approximately 100-fold increased fitness compared to the day 3 adapted isolates (*p* < 0.001) (Fig. 1B).

To determine if the increased fitness of the adapted isolates was due to a growth advantage, the in vitro CI in LB medium was investigated using the ∆*lacZ* isogenic strain. The CI for the day 3 adapted isolates was ~1.0, while the day 45 and 90 adapted isolates showed a tenfold increase in fitness (Fig. 1C) (*p* < 0.001). That the CI for intracellular survival of the adapted isolates was much greater compared to growth in LB, suggests there are additional adaptive traits allowing for the increased intracellular survival. Thus, long-term adaptation with amoeba results in *V. cholerae* isolates with increased fitness when cultured in LB and when incubated intracellularly in amoeba.

### Altered phenotypes of amoeba-adapted isolates

To elucidate the factors contributing to the increased fitness of the adapted isolates, phenotypes such as motility, biofilm formation, hemolysin and protease production were evaluated. Results showed that the motility of the adapted and non-adapted isolates decreased over time. The motility of the day 3 adapted and non-adapted isolates did not differ significantly (*p* = 0.67; mean diameter ~14 mm), while the motility of 45 and 90 day adapted isolates were significantly decreased compared to the respective non-adapted isolates (*p* < 0.001; mean 2.4 and 2.7 mm, respectively) (Fig. 1D). There was no significant difference in biofilm formation for the day 3 adapted and non-adapted isolates (*p* = 0.37) (Fig. 1E). However, the biofilm biomass of adapted isolates from days 45 and 90 was significantly reduced to 40.8% (*p* < 0.001) and 54.9% (*p* < 0.001) respectively, compared to the non-adapted isolates.

Long-term adaptation also led to significant decreases in haemolytic activity of amoeba-adapted *V. cholerae* (Fig. 1F). The haemolytic activity of the day 3 adapted and non-adapted isolates did not differ significantly (*p* = 0.31). However, the haemolytic activity of adapted isolates from days 45 and 90 decreased to 30.4% (*p* < 0.001) and 43.1% (*p* < 0.001), respectively, compared to non-adapted isolates. In contrast to the results observed for haemolytic activity, long-term co-adaptation of *V. cholerae* with amoeba resulted in increased proteolytic activity (Fig. 1G). The protease activity of adapted and non-adapted isolates of *V. cholerae* from day 3 did not differ significantly (*p* = 0.33), while it significantly increased for adapted isolates from days 45 and 90 to 37.1% (*p* < 0.001) and 59.4% (*p* < 0.001), respectively. Thus, the amoeba-adapted isolates exhibit a temporal change in phenotypes with decreases in motility, biofilm formation and hemolysin activity while exhibiting increased protease activity.

### Extended amoeba predation results in mutations in a conserved region of the master regulator *flrA*

Populations were sequenced to identify the range of variants (polymorphisms in genomes) that arose during the evolution experiment. Population sequencing at multiple time points allowed us to follow the trajectories of allelic diversity (mutations) to elucidate evolutionary dynamics. Single isolates were also sequenced to establish relationships between dominant alleles and to examine whether the single isolates carried the dominant mutations identified in the population data. We compared both datasets at corresponding time points and found correlation between them.

**Fig. 2:**
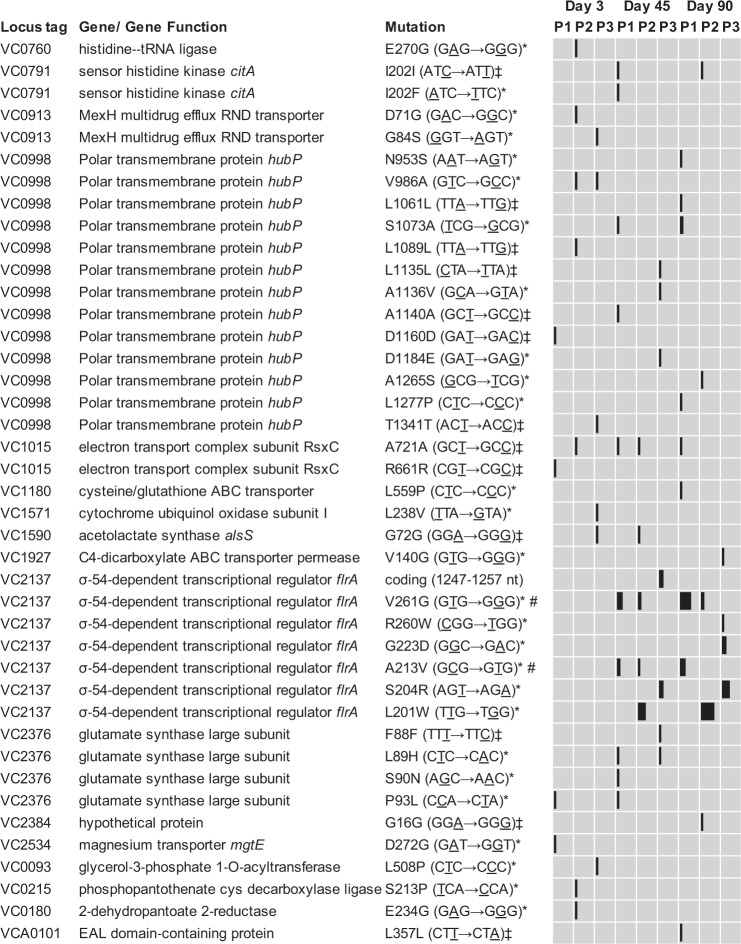
Unique mutations in coding regions of adapted populations. The locus tag and name of the affected gene are indicated in the first and second columns, respectively. The third column shows the amino acid change and its position in the protein with the affected nucleotide responsible for the amino acid change underscored in the parentheses. The black horizontal bars of the heatmap represent the frequency of respective mutations found in each population (P1, P2, P3) with value ranges from 10 to 100%. The symbols represent as follows *non-synonymous mutation, ^‡^synonymous mutation, # same mutation also found in single clone at 100% frequencies.

To identify underlying genetic changes that lead to the altered phenotypes, we analysed the genomic sequences of populations of adapted and non-adapted *V. cholerae*. A total of 144 and 146 different mutations (nsSNPs and sSNPs) and INDELs were observed in coding regions of adapted and non-adapted populations affecting 37 and 35 genes, respectively. Twenty-five mutated genes were common in both populations while 12 and 10 genes were unique to adapted and non-adapted populations, respectively (Supplementary Tables 2,  3). The *flrA* gene (VC2137) was the only gene that was consistently mutated in day 45 and 90 replicate adapted populations (6 nSNPs and 1 INDEL). The unique mutations that were detected in the coding regions in amoeba-adapted populations but not in non-adapted populations are presented in Fig. 2. Overall, most of the mutations fluctuate throughout the experiment and ultimately are lost in both adapted and non-adapted populations with very few of the mutations that appeared in the day 45 adapted populations persisting in day 90 adapted populations. Notably, four nsSNPs (L201W at 41.7%, S204R at 21.9%, A213V at 16.7% and V261G at 29.5% frequency) in the flagellar transcriptional master regulator, *flrA*, (VC2137) appeared in the day 45 adapted populations. The same four mutations also persisted in the day 90 adapted populations and the mutational frequencies increased to L201W at 74.3%, S204R at 46.6%, A213V at 29.2% and V261G at 63.3%.

**Fig. 3:**
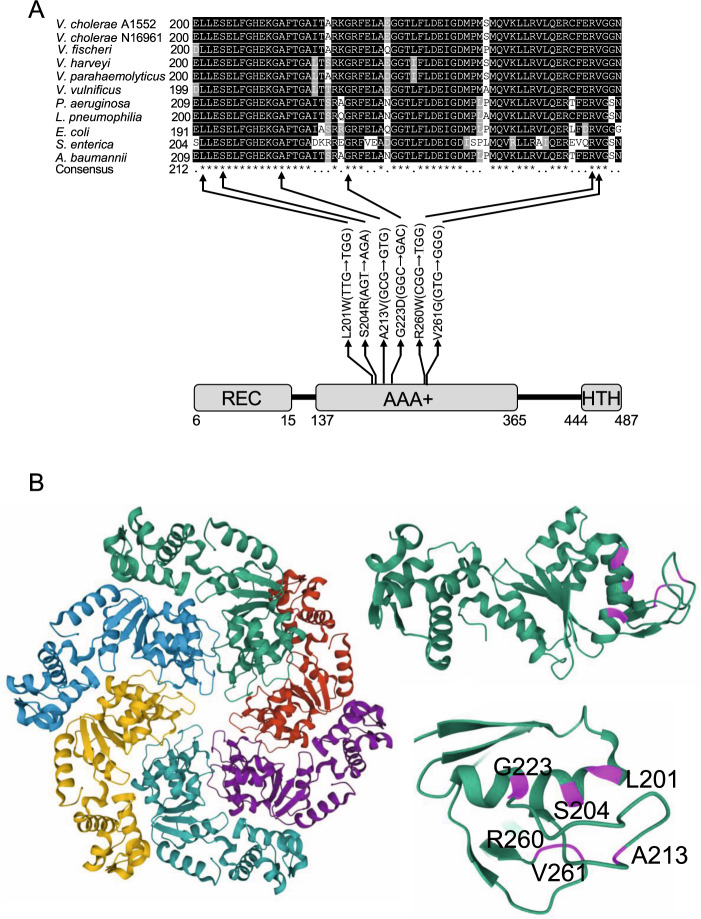
Schematic representation of the non-synonymous mutations affecting conserved regions of the FlrA protein. **A** The FlrA protein has three domains as indicated, an N-terminal signal receiver (REC) domain, a central ATPase-Associated domain with diverse cellular Activities (AAA+) and a C-terminal DNA binding helix turn helix (HTH) domain. Positions of the mutations in the central domain are indicated with respective amino acid and nucleotide base substitution in the codon. Affected amino acids in the sequence alignment of FlrA protein are indicated by black arrows. Protein sequences were retrieved from the NCBI protein database—*V. cholerae* A1552 (AUR70352), *V. cholerae* N16961 (NP_231768), *V. fischeri* (WP_011262363), *V. parahaemolyticus* (WP_025525752), *V. vulnificus* (WP_039545791), *P. aeruginosa* (NP_249788), *L. pneumophila* (WP_027221215), *E. coli* (MHO05571), *S. enterica* (WP_064013385), *A. baumannii* (SCY06189). **B** Structural model of FlrA generated from the crystal structure of the *P. aeruginosa* FleQ AAA+ domain is shown (5EXP). The monomers of the hexametric assemblies of the AAA+ domains of FleQ are shown in different colours (left). One monomer is shown with amino acid residues affected by nsSNPs highlighted in magenta (top right) and close-up view of the denoted residues is shown (bottom right).

Consistent with the population sequencing, analysis of genomes of single isolates revealed two nsSNPs (A213V and V261G) in *flrA* in the adapted isolates from days 45 and 90. These two mutations occurred at 100% frequency in the adapted isolates (Supplementary Data 3 and Supplementary Fig. 2). Unique and common genes that were mutated in adapted and non-adapted isolates are listed in Supplementary Tables 4, 5, respectively. Analysis of the mutations in *flrA* revealed that the central ATPase-associated domain with diverse cellular activities (AAA+) harbours all of the nsSNPs, while one deletion occurred in the flanking region of the central and C-terminal DNA binding helix turn helix (HTH) domain (Fig. 3A). FlrA protein sequences from different species of *Vibrio* and other Gram-negative bacteria were aligned (Fig. 3A) and show that all of the amino acids affected by the nsSNPs are highly conserved between species and in other Gram-negative bacteria (Supplementary Fig. 3). Structural modelling of the AAA+ domain with the FlrA homologue FleQ in *P. aeruginosa* revealed that all the observed nsSNPs lie in close proximity to each other (Fig. 3B). The sequence analysis of populations and single isolates showed that *flrA* is the only gene which was mutated in amoeba-adapted *V. cholerae* (days 45 and 90) but not in non-adapted *V. cholerae*.

### Changes in virulence-associated phenotypes, increased intracellular survival and fitness are due to *flrA* mutations

**Fig. 4:**
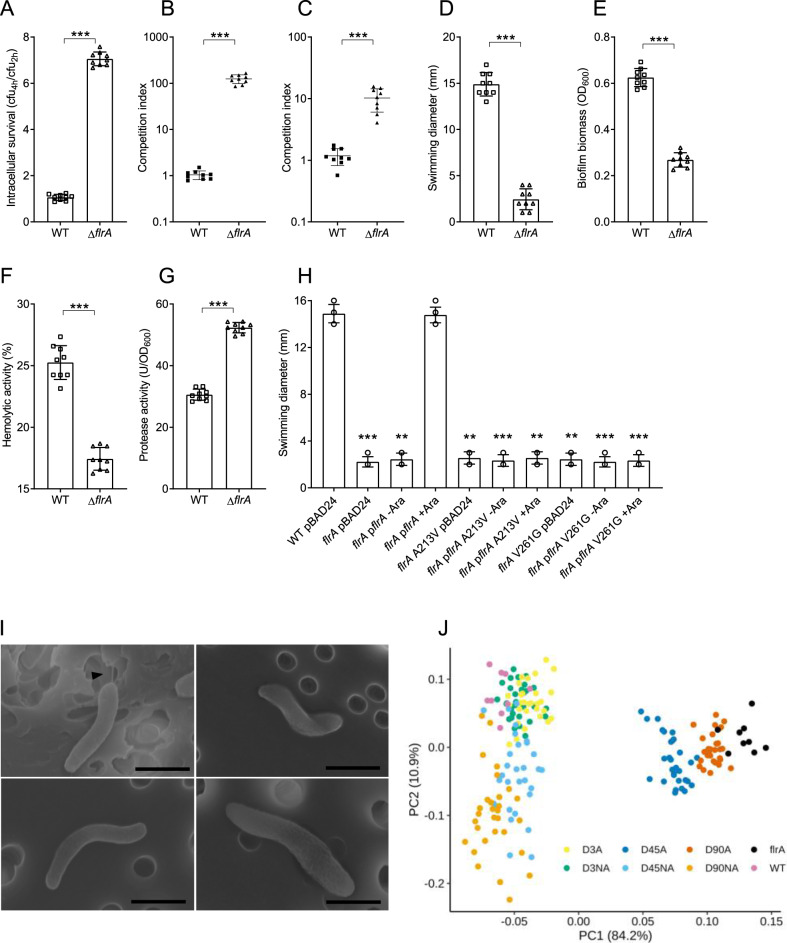
Altered phenotypes in adapted isolates are due to the mutation in *flrA*. **A** Intracellular survival of the wild type and ∆*flrA* in *A. castellanii* calculated by dividing the number of bacteria that were detected at 4 h by the 2 h samples. **B** CI of the wild type and ∆*flrA* in *A. castellanii* and LB (**C**) calculated by dividing the output ratio (WT/∆*lacZ*) after incubation corrected by the input ratio. **D** Swimming motility of the wild type and ∆*flrA* expressed as diameter (mm) of the zone travelled from point of inoculation by bacteria grown overnight on semi-solid LB agar plates. **E** Biofilm biomass of the wild type and ∆*flrA* quantified by CV staining. **F** Haemolytic activity of the wild type and ∆*flrA* expressed as percent haemolysis of 1% sheep erythrocyte suspension. **G** Protease activity in culture supernatants of the wild type and ∆*flrA* measured by azocasein hydrolysis assay. **H** Swimming motility of the wild type and ∆*flrA* complemented with either pBAD24 or pBAD24 containing wild type *flrA* and/or mutated copies of the *flrA* (A213V and V261G). Values are expressed as diameter (mm) of the zone travelled from point of inoculation by bacteria grown overnight on semi-solid LB agar plates containing ampicillin and with or without arabinose. All of the data are from nine independent biological replicates and are shown as the mean ± standard deviation. Statistical significance was determined by two-tailed, non-parametric Mann–Whitney test (**A**–**G**), one-way ANOVA and Kruskal–Wallis test (**H**) and is indicated by ^∗∗∗^*p* < 0.001. **I** Scanning electron micrograph showing presence or absence of flagellum on *V. cholerae*. Wild type A1552 (top left), A1552 Δ*flrA* (top right), A1552 *flrA* A213V (Bottom left), A1552 *flrA* V261G (Bottom right). Presence of flagella in wild type image indicated with black arrow. Scale bars: 1 µm. **J** Principal component analysis (PCA) on the changes in the four virulence phenotypes (motility, biofilm, hemolysin and protease) across adapted isolates, non-adapted isolates, wild type and ∆*flrA* mutant.

To examine whether genetic mutations in *flrA* explain the observed changes in virulence factor production, increased intracellular survival and improved fitness of the 45 and 90 day adapted isolates, a *flrA* deletion mutant (∆*flrA*) in the ancestral background was generated. Interestingly, all of the phenotypic features that were observed in the adapted isolates were also observed in the ∆*flrA* mutant. The intracellular survival of the ∆*flrA* was approximately seven times greater than the WT and the mean survival rate of the non-adapted isolates (Fig. 4A). The CI of the ∆*flrA* was 100-fold greater compared to the WT in amoeba (Fig. 4B) and tenfold greater in LB (Fig. 4C). The ∆*flrA* had a 7.5-fold reduction in swimming motility (diameter of 2 mm) (Fig. 4D), a 55% reduction in biofilm biomass (Fig. 4E) and a 30% reduction in haemolysin activity compared to the WT (Fig. 4F). In contrast, the average protease activity for ∆*flrA* was 70% higher compared to WT (Fig. 4G). PCA of the changes in the four phenotypes (motility, biofilm, hemolysin and protease) reveals divergent patterns of the adapted isolates. The day 3 adapted isolates clustered with all the non-adapted isolates, while days 45 and 90 adapted isolates clustered with the ∆*flrA* (Fig. 4J).

To further confirm the mutations that arose during amoeba predation in *flrA* leads to all of the observed phenotypes, we targeted two nsSNPs in the *flrA* gene, A213V and V261G. These two mutations appeared in single isolates from the adapted populations. The WT copy of *flrA* as well as the mutated copies (A213V and V261G) were expressed in a ∆*flrA* background under the control of the arabinose inducible promoter of the expression vector pBAD24. To compare the motility of the complemented clones of the ∆*flrA*, an empty vector control was also tested. As expected, *flrA* complementation and induction with arabinose restores motility of the ∆*flrA* to WT levels while the expression of the mutated *flrA* alleles does not (Fig. 4H) To determine the effect of the point mutations in *flrA* on flagellum formation, adapted isolates harbouring A213V and V261G mutations in *flrA* were analysed under scanning electron microscopy. Image analysis confirmed a lack of flagellum on adapted isolates with the *flrA* point mutations (Fig. 4I). Hence, the alanine at 213 and valine at 261 are two essential residues in FlrA required for flagella production in *V. cholerae*.

### Adaptation leads to enhanced colonisation of an aquatic host

**Fig. 5:**
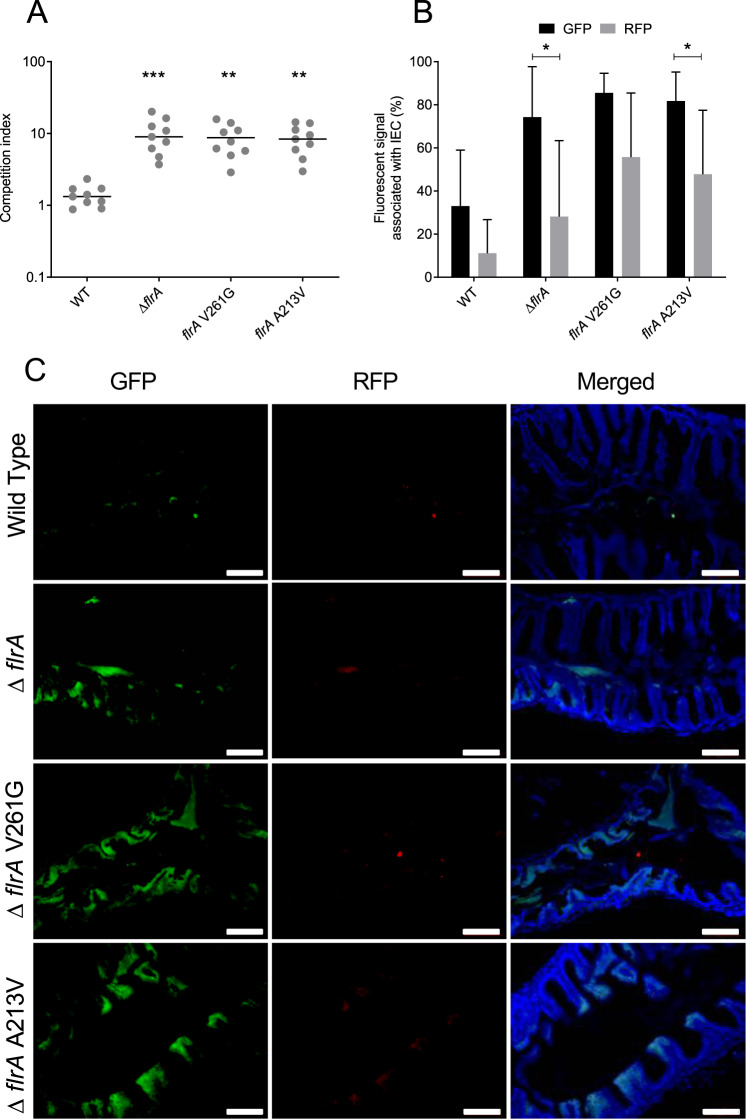
Enhanced colonisation of adapted isolates in a zebrafish infection model. **A** CI of adapted isolates with mutations in *flrA* in zebrafish infection model was calculated by dividing the output ratio of test strain (e.g. *flrA* mutants) to ∆*lacZ* after infection corrected by the input ratio. Data are from nine independent biological replicates and are shown as the median. Statistical significance was determined by one-way ANOVA and Kruskal–Wallis test and is indicated by ^∗∗∗^*p* < 0.001 and ^∗∗^*p* < 0.01. **B** Quantification of bacterial fluorescence associated with intestinal epithelial cells of zebrafish after infection. Data are presented as percentages of GFP (test strain) and RFP (∆*lacZ* mutant) signal quantified by fluorescent pixel count of tissue sections in ImageJ. Statistical significance was determined by one-way ANOVA and Tukey’s multiple comparison test and is indicated by ^∗^*p* < 0.05. **C** Colonisation of adapted isolates in adult zebrafish intestine. The four panels show representative fluorescence microscopy images of intestinal epithelial cell of adult zebrafish colonised with the indicated bacteria tagged with GFP and ∆*lacZ* strain tagged with RFP. Scale bars: 100 µm.

Since the adapted isolates showed differences in production of multiple virulence-related traits, we hypothesised that they were primed for colonisation of higher eukaryotic organisms. To test this, the adult zebrafish model was used as this model supports a natural route of transmission and has been a suitable model for studies of colonisation and transmission of *V. cholerae* [47–49]. To measure the colonisation of the adapted isolates harbouring mutations in the *flrA* (A213V and V261G) relative to the ∆*lacZ* strain competition assays were performed. The CI was calculated from the cells recovered from each fish intestine after 24 h of infection. The results indicated that the ∆*flrA* colonised adult zebrafish 10-fold better than the ∆*lacZ* strain. The strains harbouring point mutations (A213V and V261G) in *flrA* colonised similarly to the ∆*flrA* (Fig. 5A). Zebrafish were infected with GFP-tagged test strains (Wild type, ∆*flrA, flrA* A213V, *flrA* V261G) and an RFP-tagged ∆*lacZ* strain. Imaging revealed greater binding to fish intestinal epithelial cells of *V. cholerae flrA* mutants compared to the ∆*lacZ* strain (Fig. 5B, C). The proportion of GFP and RFP fluorescence associated with fish intestinal epithelium cells recapitulated the trends seen in the CFU recovery assay. Hence, adaptation in amoeba increases the potential of *V. cholerae* to colonise higher eukaryotic organisms in the natural environment and contributes to the persistence and dissemination of the bacterium.

## Discussion

The results described here have important implications for understanding the molecular mechanisms of bacterial adaptation and persistence in the environment. During intense predation pressure by free-living protozoa, *V. cholerae* is able to survive inside protozoan hosts. The results presented here show that this is achieved through a trade-off among multiple virulence traits that ultimately lead to increased fitness. The loss of motility may protect *V. cholerae* by reducing the frequency of contact with predators. It has been previously reported that motile bacteria experience increased rates of contact with raptorial feeding protists and hence are more susceptible to being ingested than non-motile cells [22, 23]. In addition, the flagellin protein functions as a pathogen-associated molecular pattern that binds to pattern-recognition receptors on a variety of defence cells, including macrophage and amoeba, thereby activating phagocytosis [50, 51].

Other reports suggest that the production of protease is critical for neutralisation of the HlyA haemolysin [52]. The haemolysin (HlyA) causes lysis of amoeba and by degrading the haemolysin, premature lysis of the amoeba host is prevented, hence ensuring a successful replication niche [19]. Here, we report that co-adaptation with amoeba led to an increase in protease activity and a concomitant decrease in haemolysin activity, further supporting the hypothesis that expression of haemolysin in amoeba is not advantageous for *V. cholerae*. The loss of motility has been shown to have a negative impact on biofilm formation, and it is likely that the decrease in immunogenic impacts of flagella outweigh the benefits during long-term co-adaptation, and is thus the result of a trade-off to ensure successful replication within the amoeba host. Further, as amoeba selectively graze on biofilm cells the long-term adaptation leads to less biofilm, further reducing predation pressure. The increase in intracellular survival and competitive fitness of adapted isolates in amoeba are the effect of synergistic actions of multiple traits in the adapted isolates.

Genome analysis of the amoeba-adapted *V. cholerae* populations and isolates revealed that the mutations in *flrA* were responsible for the increased fitness and changes in phenotypic traits. FlrA is the main regulator of the flagellar regulon and its inactivation leads to loss of flagellar synthesis [53, 54]. The regulator is widespread in bacteria and regulates diverse functions including motility, biofilm formation, virulence factor expression and sensing of small molecules [55–57]. The absence of a flagellum hampers early stages of biofilm formation in *V. cholerae* [58]. Consistent with our study, Syed et al. showed that absence of *flrA* increases the expression of the secreted proteases, HapA and PrtV, and the hemolysin HlyA [54]. It is likely that increased protease production in *V. cholerae* degrades the secreted haemolysin explaining our observations in the late-stage amoeba-adapted isolates [52]. The phenotypes observed in the late-stage amoeba-adapted isolates are an indication of increased HapR activity. HapR is the quorum-sensing master regulator in *V. cholerae* which positively regulates production of protease while negatively regulates biofilm formation [59]. Future studies will be needed to identify the putative link between *flrA* and *hapR*. However, it has been previously shown that FliA, a downstream target of FlrA, represses *hapR* expression [60]. Hence, in absence of FlrA the repression of *hapR* is likely be relieved thus increased proteases expression and decreased biofilm formation in late-stage adapted isolates might be due to the increased HapR activity.

Significantly, this study demonstrated that selection of multiple virulence-related traits in *V. cholerae* during adaptation with an amoebal host leads to increased colonisation in the zebrafish model of infection. As fish are potential reservoirs of *V. cholerae*, the increased colonisation may further contribute to their persistence in the environment and may play an important role in dissemination [61–63]. Our results support the hypothesis that adaptation to amoeba drives selection of multiple phenotypic traits which improved fitness and increased colonisation in a higher eukaryotic host. Surprisingly, point mutations in a single gene, *flrA*, associated with regulation of flagella, recapitulated the changes in phenotypes observed in adapted isolates as well as in colonisation of zebrafish. This highlights how predation can select for strains with enhanced capacity to colonise other hosts, which may be the result of only one or two nucleotide changes. Future studies will be needed to identify similar mutations in natural isolates of *V. cholerae*. However, this study provides a predictive model where increased microbial interaction due to global warming may lead to such outcomes as rises in temperature drives increased microbial abundance and thus *Vibrio* spp. in the marine environment [64].

A previous study showed that the absence of *flrA* in *V. cholerae* leads to colonisation defects in the infant mouse colonisation model, which is the gold standard model for cholera infection [53]. There are also reports showing that virulence factors that are involved in colonisation of zebrafish differ from those that are important in the infant mouse model [48]. Thus, the varied factors important for colonisation in these two model systems might indicate that a trade-off in colonisation factors would lead to increased colonisation of zebrafish while decreasing colonisation in the infant mouse model. This would result in the variants being less successful in the human context while increasing the potential for transmission and dissemination in the environment. Indeed, there have been reports showing predation can lead to decreased virulence [10, 65]. Together these phenotypic and genotypic changes contribute to our understanding of defensive and adaptive mechanisms of *V. cholerae* exhibits in the environment under predatory pressure.

## Supplementary information

Supplementary Information

Dataset 1

Dataset 2

Dataset 3

Dataset 4

## Data Availability

Sequence data generated in this study are available from the NCBI Sequence Read Archive with accession number PRJNA685017.
