# Supplementary material for: Adaptation to an amoeba host drives selection of virulence-associated traits in Vibrio cholerae
Source: ISME J. 2021 Oct 15;16(3):856–67. doi: 10.1038/s41396-021-01134-2 (PMC8857207; doi:10.1038/s41396-021-01134-2)
Supplement: Supplementary file 1 — Supplementary Information [file 41396_2021_1134_MOESM1_ESM.pdf]

Supplementary information

Adaptation to an amoeba host drives selection of virulence-associated traits in *Vibrio cholerae*

M. Mozammel Hoque[1], Parisa Noorian[1], Gustavo Espinoza-Vergara[1], Pradeep Manuneedhi Cholan[2,3], Mikael Kim[4], Md Hafizur Rahman[5], Maurizio Labbate[5], Scott A. Rice[1,6], Mathieu Pernice[4], Stefan H. Oehlers[2,3], Diane McDougald[1,6*].

[1]The iThree Institute, University of Technology Sydney, Sydney 2007, Australia.

[2]Tuberculosis Research Program at the Centenary Institute, The University of Sydney, Camperdown NSW 2050, Australia.

[3]The University of Sydney, Faculty of Medicine and Health & Marie Bashir Institute, Camperdown NSW 2050, Australia.

[4]Climate Change Cluster, University of Technology Sydney, Sydney, New South Wales, Australia.

[5]School of Life Sciences, Faculty of Science, University of Technology Sydney, Sydney, New South Wales, Australia.

[6]Singapore Centre for Environmental Life Sciences Engineering, Nanyang Technological University, Singapore 637551.

*Correspondence to: diane.mcdougald@uts.edu.au

Contents:

Supplementary Figures 1-3.

Supplementary Table 1-5

Supplementary Data Legends 1-4

Supplementary references

Supplementary Figures

Supplementary Fig. 1

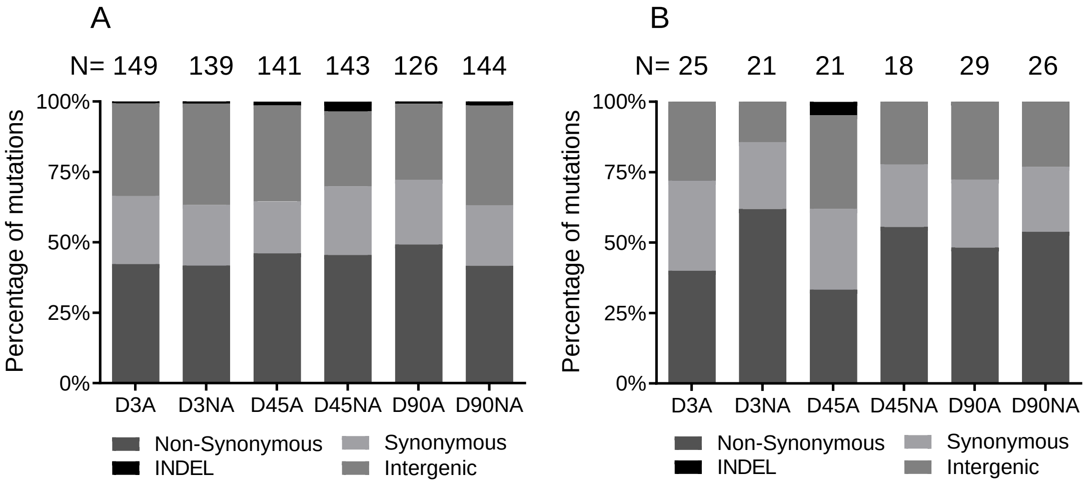

Supplementary Fig. 1. Mutations occurring in adapted and non-adapted populations (A) and isolates (B). Shaded bars show the distribution of different types of mutations in the adapted (A) and non-adapted (NA) populations at three different time points (D3, D45 and D90). The total number of mutations (N) are shown above each column. Base substitutions are shown as nonsynonymous, synonymous, intergenic mutations and indel.

Supplementary Fig. 2

Locus tag	Gene/ Gene function	Mutation
VC0744	protein translocase subunit	E101G (GAG→GGG)
VC0766	exodeoxyribonuclease	S148P (TCA→CCA)
VC0778	siderophore transporter permease	E246G (GAG→GGG)
VC0791	sensor histidine kinase	Y183S (TAC→TCC)
VC0905	met transporter binding protein	N58K (AAT→AAA)
VC0913	multidrug efflux RND transporter	N78K (AAT→AAA)
VC0998	Polar transmembrane protein	D948E (GAT→GAG)
VC0998	Polar transmembrane protein	D996E (GAT→GAG)
VC0998	Polar transmembrane protein	T1033A (ACA→GCA)
VC0998	Polar transmembrane protein	V1094A (GTC→GCC)
VC0998	Polar transmembrane protein	T1095A (ACG→GCG)
VC0998	Polar transmembrane protein	V1125A (GTA→GCA)
VC0998	Polar transmembrane protein	S1145F (TCT→TTT)
VC0998	Polar transmembrane protein	L1185P (CTC→CCC)
VC0998	Polar transmembrane protein	V1217A (GTC→GCC)
VC0998	Polar transmembrane protein	T1229S (ACT→AGT)
VC0998	Polar transmembrane protein	P1238T (CCC→ACC)
VC1205	imidazolonepropionase	P60Q (CCG→CAG)
VC1205	imidazolonepropionase	P60T (CCG→ACG)
VC1205	imidazolonepropionase	T59K (ACA→AAA)
VC1205	imidazolonepropionase	V58F (GTC→TTC)
VC1205	imidazolonepropionase	L57* (TTA→TGA)
VC1205	imidazolonepropionase	G55V (GGC→GTC)
VC1451	multifunctional autoprocessing toxin	V1112G (GTA→GGA)
VC1455	XRE family transcriptional regulator	A68D (GCT→GAT)
VC1798	ATPase AAA	E292G (GAG→GGG)
VC1821	PTS fructose transporter IIC	A190S (GCG→TCG)
VC1821	PTS fructose transporter IIC	I191F (ATT→TTT)
VC1821	PTS fructose transporter IIC	G193D (GGT→GAT)
VC1821	PTS fructose transporter IIC	G194D (GGT→GAT)
VC1821	PTS fructose transporter IIC	F198Y (TTC→TAC)
VC1821	PTS fructose transporter IIC	D199E (GAT→GAG)
VC2021	beta-ketoacyl-ACP reductase	I244L (ATC→CTC)
VC2137	σ-54-dependent transcriptional regulator *flrA*	V261G (GTG→GGG)
VC2137	σ-54-dependent transcriptional regulator *flrA*	A213V (GCG→GTG)

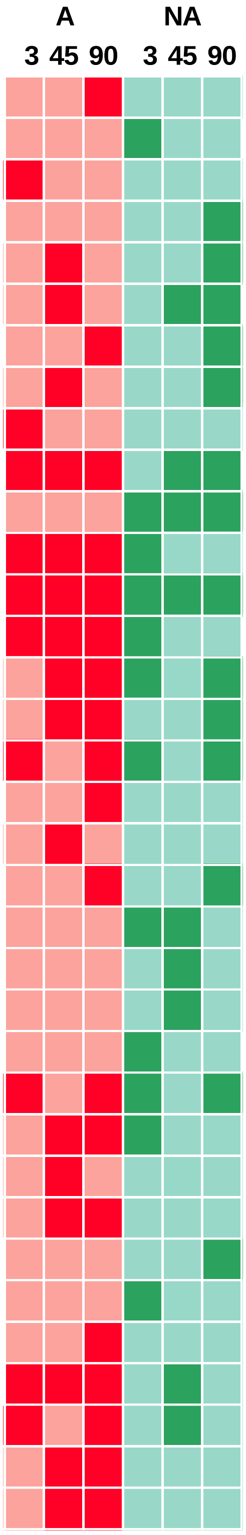

Supplementary Fig. 2. Non-synonymous mutations in coding regions of adapted and non-adapted isolates. The locus tag and name of the affected gene are indicated in the first and second columns, respectively. The third column shows the type of amino acid changes with position in the

protein with affected base changes highlighted with underscore. The heatmap shows the presence (deep color) and absence (shaded color) of nsSNPs found in coding region of adapted and non-adapted isolates respectively in any of the three replicates from three different time points day 3, 45 and 90. The symbols represent as follows A, Adapted and NA, Non-adapted.

Supplementary Fig. 3

```
V. cholerae_A1552    1 -MQSLAKLLVIEDDAAIRLNLSVILEFVGEQCEVIESTQIDQ-IN---WSAVWGGCILGS
V. cholerae_N16961   1 -MQSLAKLLVIEDDAAIRLNLSVILEFVGEQCEVIESTQIDQ-IN---WSAVWGGCILGS
V. fischeri          1 -MQGLAKTLIIEDDAQSRHNLKVILEFIGEHCHAISASELST-FE---WSEPWSACFLGQ
V. harveyi           1 -MQGLAKLLVIDDDAPSRLNISNILEFVGESCEAVCSDQIGD-VD---WSAVWSGCIVGN
V. parahaemolyti     1 -MQGLAKLLVIDDDASSRLNLSNILEFVGESCEAVGSEQIGD-VD---WSSVWSGCIVGN
V. vulnificus        1 -MQGLAKLLVIEDDEANRLNLRNILEFVGESCEALRSDQIEN-AD---WSKIWSGVIVGF
P. aeruginosa        1 -MWRETKLLLIDDNLDRSRDLAVILNFLGEDQLTCNSEDWREVAAGLSNSREALCVLLGS
L. pneumophila       1 -MRSNDRIYIIDDNKERCDKLRTIFDFIGQSSEVTQYDNWQLSVN-----PNPSVIVLGA
E. coli              1 ---MAMGVLIVDADAQRRQQLGTVLSFMGIPWQEIAESELDVAIE---TSGPLQGVLAGA
S. enterica          1 MIRGKIDILVVDDDVSHCTILQALFRGWGYNVALAYSGH-DALAQ--VREKVFDLVLCDV
A. baumannii         1 -MWRETKLLLIDDNLDRSRDLAVILNFLGEDQLTCNSEDWREVAAGLSNSREALCVLLGS
consensus            1  .. ..........  . ..  .....*. .. . ....   .    .. .. .....

V. cholerae_A1552   56 LR-GQALSEQLIQSLTKANHIPLLVANK---QPYSLEE-FPNYVGELDFPLNYPQLSDAL
V. cholerae_N16961  56 LR-GQALSEQLIQSLTKANHIPLLVANK---QPYSLEE-FPNYVGELDFPLNYPQLSDAL
V. fischeri         56 VS-DQALKDIIKSSLVVHNHIPVIMLAG---AMHDVEE-LTNYVGELNQPVNYPQLTDAL
V. harveyi          56 IS-AGHSATAVMAHLNEAYHIPLLVMGS---FPLPVDD-LPNFVGELEQPLNYPQLSEAL
V. parahaemolyti    56 IS-AGRAATAVMARLNDAYHIPLLVLGS---FPLPVDD-LPNFVGELEQPLNYPQLSEAL
V. vulnificus       56 VD-N-KSITTMMAKLNSAHHIPLLVLGD---FSHPVEH-LPNLIGELEFPLNYPQLSEAL
P. aeruginosa       60 VESKGGAVELLKQLASWDEYLPILLIGEPAPADWPEEL-RRRVLASLEMPPSYNKLLDSL
L. pneumophila      55 CTSFEKTMDELDALVKRFPKIPILVIDAV--LSTSECA-DRNVVDCLSFPFSYAKMLKSL
E. coli             55 LT-GRPLDE----LLTLFPRIPFLSLVP---ADFP----NSNFIGVAEAPFTYESLIRYL
S. enterica         58 RMAEMDGIATLKEIKALNPAIPILIMTAFSSVETAVEALKAGALDYLIKPLDFDRLQETL
A. baumannii        60 VESKGGAVELLKQLASWDEYLPILLIGEPAPADWPEEL-RRRVLASLEMPPSYNKLLDSL
consensus           61 .        . .   .    ..*.....       ...    ....... *.........*

V. cholerae_A1552  111 RHCKEFLGRKGF-QVLATARKNTLFRSLVGQSMGIQEVRHLIEQVSTTEANVLILGESGT
V. cholerae_N1696  111 RHCKEFLGRKGF-QVLATARKNTLFRSLVGQSMGIQEVRHLIEQVSTTEANVLILGESGT
V. fischeri        111 RHCQEFMGRRGL-EVPHLGRKNTLFRSMVGQSTAISTVRHLIEQVSGTEASVLVLGESGT
V. harveyi         111 RHCKDFLGRKGV-NVVASARKNTLFRSLVGQSRGIQEVRHLIEQVSGTEANVLILGESGT
V. parahaemolyti   111 RHCKDFLGRKGV-NVVASARKNTLFRSLVGQSRGIQEVRHLIEQVSGTEANVLILGESGT
V. vulnificus      110 RHCKEFLGRKGV-NVVASARKNTLFRSLVGQSLGIQEVRHLIEQVAATEANVLILGESGT
P. aeruginosa      119 HRAQVYREMYDQARERGRSREPNLFRSLVGTSRAIQQVRQMMQQVADTDASVLILGESGT
L. pneumophila     112 HRCQIAKEAVKFIT--AGTHKTPLFRSLVGNSEGIRQVRKLIEQVADTEASVLILGESGT
E. coli            103 HFCQAFVSLHPR-QQT-HDKGQALLRLLVGKGRGIQEVRRLISQVAETDANVLILGESGT
S. enterica        118 EKALAHTRETGA----ELPSASAAQFGMIGSSPAMQHLLNEIAMVAPSDATVLIHGDSGT
A. baumannii       119 HRAQVYREMYDQARERGRSREPNLFRSLVGTSRAIQQVRQMMQQVADTDASVLILGESGT
consensus          121 ... .......   .. . ...........*.. ...........*. ..*.**..*.***

V.cholerae_A1552 170 GKEVVARNIHYHSGRRNGPFVPINCGAIPAELLESELFGHEKGAFTGAITARKGRFELAE
V.cholerae_N1696 170 GKEVVARNIHYHSGRRNGPFVPINCGAIPAELLESELFGHEKGAFTGAITARKGRFELAE
V.fischeri       170 GKEVVARNLHYHSPRRKGPFVPVNCGAIPPDLLESELFGHEKGAFTGAITARKGRFELAQ
V.harveyi        170 GKEVVARNIHYHSAYRNGPFVPINCGAIPPELLESELFGHEKGAFTGALTSRKGRFELAE
V.parahaemolyti  170 GKEVVARNIHYHSSYRNGAFVPINCGAIPPELLESELFGHEKGAFTGALTARKGRFELAD
V.vulnificus     169 GKEVVARNIHYHSSRRNGPFVPINCGAIPPDLLESELFGHEKGAFTGALTTRKGRFELAE
P.aeruginosa     179 GKEVVARNLHYHSKRREGPFVPVNCGAIPAELLESELFGHEKGAFTGAITSRAGRFELAN
L.pneumophila    170 GKEVVARNIHSLSSRANKPFVPINCGAIPGELLESELFGHEKGAFTGAITSRQGRFELAN
E.coli           161 GKEVVARAIHELSSRCAGPFVPINCGAIPAELLESELFGHEKGAFTGAIASRRGRFELAQ
S.enterica       174 GKEIVARALHACSARSDKPLVTLNCAALNESLLESELFGHEKGAFTGADKRREGRFVEAD
A.baumannii      179 GKEVVARNLHYHSKRREGPFVPVNCGAIPAELLESELFGHEKGAFTGAITSRAGRFELAN
consensus        181 ***.***..*..* ......*..**.*....*****************...*.***..*.

V. cholerae A1552  230 GGTLFLDEIGDMPMSMQVKLLRVLQERCFERVGGNSTIKANVRVIAATHRNLEEMIDGQK
V. cholerae_N1696  230 GGTLFLDEIGDMPMSMQVKLLRVLQERCFERVGGNSTIKANVRVIAATHRNLEEMIDGQK
V. fischeri        230 GGTLFLDEIGDMPMPMQVKLLRVLQERCFERVGGNQSIRADVRIVAATHRNLDEMIEKGT
V. harveyi         230 GGTIFLDEIGDMPMSMQVKLLRVLQERCFERVGGNTTIKVNVRVVAATHRNLEGMIDDGT
V. parahaemolyti   230 GGTIFLDEIGDMPMSMQVKLLRVLQERCFERVGGNSTIKVNVRVVAATHRNLESMIEEGT
V. vulnificus      229 GGTLFLDEIGDMPMAMQVKLLRVLQERCFERVGGNTTIKANVRIIAATHRNLETMIDDEK
P. aeruginosa      239 GGTLFLDEIGDMPLPMQVKLLRVLQERTFERVGSNKTQNVDVRIIAATHKNLEKMIEDGT
L. pneumophila     230 GGTLFLDEIGDMPLAMQVKLLRVLQERCFERVGSNKSIEVNVRIIAATHRNLEIAIQEGK
E. coli            221 GGTLFLDEIGDMPLPMQVKLLRVLQERLFDRVGGGKPIQADVRIIAATHRDLEVMIRTQG
S. enterica        234 GGTLFLDEIGDISPLMQVRLLRAIQEREVQRVGSNQTISVDVRLIAATHRDLAEEVSAGR
```

```
A. baumannii       239 GGTLFLDEIGDMPLPMQVKLLRVLQERTFERVGSNKTQNVDVRIIAATHKNLEKMIEDGT
consensus          241 ***.*******... ***.***..***...***.. .....**..****..*. .....

V. cholerae_A1552  290 FREDLYYRLNVFPIEMPALRDRIDDIPLLLQELMTRMEAEGAQP-ICFTPRAINSMMEHD
V. cholerae_N1696  290 FREDLYYRLNVFPIEMPALRDRIDDIPLLLQELMTRMEAEGAQP-ICFTPRAINSMMEHD
V. fischeri        290 FREDLYYRLNVFPIEMPALRERKQDIPLLLQELLARMEAEGSTR-VRLTPRAVNSLMEHH
V. harveyi         290 FREDLFYRLNVFPIEMPALKARKQDIPLLLQELMTRLEAEGGQP-ICFTPRAINSLMEHN
V. parahaemolyti   290 FREDLFYRLNVFPIEMPALKERKQDIPLLLQELMTRLEAEGGQP-ICFTPRAINSLMEHH
V. vulnificus      289 FREDLFYRLNVFPIEMPALKERKEDIPLLLQELMTRLQAEGGLP-ICFTPRAINSLMEHD
P. aeruginosa      299 FREDLYYRLNVFPIEMAPLRERVEDIALLLNELISRMEHEKRGS-IRFNSAAIMSLCRHD
L. pneumophila     290 FREDLFYRLNVFPIEMPPLRNRSSDLSLLFNELVSRIEGEGRPT-VRLMPDAMDALCEYS
E. coli            281 FREDLYYRLNVFPIETPPLRERVDDIPLLLQELLNRHAEQHHGI-IRLTQRAMESLMQYP
S. enterica        294 FRQDLYYRLNVVAIEMPSLRQRREDIPLLAEHFLQRFAARNRKAVKGFTPQAMDLLIHYD
A. baumannii       299 FREDLYYRLNVFPIEMAPLRERVEDIALLLNELISRMEHEKRGS-IRFNSAAIMSLCRHD
consensus          301 **.**.*****..**...*..* .*..**......*.....    . ....*........

V. cholerae_A1552  349 WPGNVRELANLVERMVILYPNSLVDVNHLPTKYRYSDIPEFQPEPSRFSSVEEQERDVLE
V. cholerae_N1696  349 WPGNVRELANLVERMVILYPNSLVDVNHLPTKYRYSDIPEFQPEPSRFSSVEEQERDVLE
V. fischeri        349 WPGNVRELANLIERLMILFPGESVDVNHLPTKYRYSDIPEFQPENYNQQSIEEQEQAALA
V. harveyi         349 WPGNVRELANLVERMVILYPNSLVDVNHLPTKYRYSDIPEFQPEGNPFTSVEEQERDVFQ
V. parahaemolyti   349 WPGNVRELANLVERMIILYPNSLVDVNHLPTKYRYSDIPEFQPEGNPFTSIEEQERDVFQ
V. vulnificus      348 WPGNVRELANLVERMVILYPNSLVDVNHLPTKYRYSDIPEFQPETSSFNSIEDQERDVLE
P. aeruginosa      358 WPGNVRELANLVERLAIMHPYGVIGVGELPKKFRHVDDEDEQ-LASSLREE-LEERAAI-
L. pneumophila     349 WPGNVRELANLVERLSILYPNGIIGKSDLPHRFQGEFKPSYPEVSGSERET---------
E. coli            340 WPGNVRELSNLLERLLILYPQQIVDVADLPSRYRLLPCE---PRDE--RLTEMDERDALA
S. enterica        354 WPGNIRELENAIERSVVLLTGEYISERELPLAIAATPIKAE-------------------
A. baumannii       358 WPGNVRELANLVERLAIMHPYGVIGVGELPKKFRHVDDEDEQ-LASSLREE-LEERAAI-
consensus          361 ****.***.*..**....... ......**..............     ........ .

V. cholerae_A1552  409 GIFAEDFNFEEPQ-EFVPDIDAPQALPPEGVNLKELLADLEVNLINQALEAQGGVVARAA
V. cholerae_N1696  409 GIFAEDFNFEEPQ-EFVPDIDAPQALPPEGVNLKELLADLEVNLINQALEAQGGVVARAA
V. fischeri        409 DMFANFSDGGIPQQGMQNAGPTSNDLPEDGLNLKEMLAEVEVNMICQALEAQNGVVARAA
V. harveyi         409 DIFSENFSFDEQN-DLDHNMNAPQGLPPEGVNLKELLADMEVNMISQALEAQGGVVARAA
V. parahaemolyti   409 DIFSEDFNFDEQS-DLDHNINAPQALPPEGVNLKELLADLEVNMISQALEAQGGVVARAA
V. vulnificus      408 DIFSESFDLATRN-NFDDHFDAPQSLPPEGVNLKELLADLEVNMINQALEAQGGVVARAA
P. aeruginosa      415 -------------NAGLPGMDAPAMLPAEGLDLKDYLANLEQGLIQQALDDAGGVVARAA
L. pneumophila     400 ---------------LLQIIKQESVANAEGIDLKEHLVKTELALISQALDESDWVVAHAA
E. coli            395 AVFQDPPE-----LDMHNSHARAMQLPEEGVNLKEMLADLEVELIRQALESQDGVVARAA
S. enterica        395 -----------------------------SSAEIQPLVDVEKEIILAALEKTGGNKTEAA
A. baumannii       415 -------------NAGLPGMDAPAMLPAEGLDLKDYLANLEQGLIQQALDDAGGVVARAA
consensus          421  .. .                    . ... .. ........*...*...* .**.........**

V. cholerae_A1552  468 DMLGMRRTTLVEKMRKYNMQR--------
V. cholerae_N1696  468 DMLGMRRTTLVEKMRKYNMQR--------
V. fischeri        469 DMLGMRRTTLVEKMRKYNLNKDDMR----
V. harveyi         468 DMLGMRRTTLVEKMRKYNLQR--------
V. parahaemolyti   468 DMLGMRRTTLVEKMRKYNLQR--------
V. vulnificus      467 DMLGMRRTTLVEKMRKYNMQR--------
P. aeruginosa      462 ERLRIRRTTLVEKMRKYGMSRRDDDLSDD
L. pneumophila     445 SYLNMRRTTLVEKMRKYGLTRPERMER--
E. coli            450 DLLGMRRTTLVEKMKKYGMSKEV------
S. enterica        426 RQLGITRKTLLAKLSR-------------
A. baumannii       462 ERLRIRRTTLVEKMRKYGMSRRDDDLSDD
consensus          481 ..*...*.**..*........
```

Supplementary Fig. 3. Amino acid sequence alignment of FlrA protein related to figure 4. Protein sequences were retrieved from NCBI protein database from *V. cholerae* A1552 (AUR70352), *V. cholerae* N16961 (NP_231768), *V. fischeri* (WP_011262363), *V. parahaemolyticus* (WP_025525752), *V. vulnificus* (WP_039545791), *P. aeruginosa*

(NP_249788), *L. pneumophila* (WP_027221215), *E. coli* (MHO05571), *S. enterica* (WP_064013385), *A. baumannii* (SCY06189) respectively. Multiple sequence alignment was done on T-coffee server and annotated using Expasy Box shade tool. Affected amino acids are highlighted with yellow shadings. Identical amino acids in all of these FlrA protein sequences are shown in the consensus.

Supplementary Table

Supplementary Table 1. List of strains, plasmids, and primers

**Organisms/Strains**	**Description**	**Reference**
Vibrio cholerae A1552	Wild type, O1, El Tor, Inaba, smooth, Rif^r	[1]
Vibrio cholerae A1552 Δ*lacZ*	In-frame deletion mutant of *lacZ* gene on wild type	[2]
Vibrio cholerae A1552 Δ*flrA*	In-frame deletion mutant of *flrA* gene on wild type	This Study
Escherichia coli DH5α	F endA1 hsdR17 supE44 thi-1 recA1 gyrA96 relA1 (argF-lacZYA) U169 (80lacM15)	Laboratory collection
E. coli BW20767	RP42tet::Mu1kan::Tn7- integrant uidA(DMlu1)::pir+ recA1 creB510 leu63 hsdR17 endA1 zbf5 thi	ATCC 47084
E. coli S17-1 λ pir	recA thi pro rK- mK+ RP4::2-Tc::MuKm Tn7 Tpr Smr λ pir	ATCC 47055
Acanthamoeba castellanii		ATCC 30234
Zebrafish (*Danio rerio*)		[3]
**Oligonucleotide/Primer**		
Primers for construction of *flrA* mutant		
flrA-up-F	GAAGAAGAACTCGACGCTCAA	This study
flrA -up-R	CCAGCCTACACGGCATCGTCCTCAATCACAAGT	This study
flrA -cat-F	GGACGATGCCGTGTAGGCTGGAGCTGCTTC	This study
flrA -cat-R	TGCGCATCTTCTCAACCATATGAATATCCTCCTTAG	This study
flrA -down-F	GAGGATATTCATATGGTTGAGAAGATGCGCAAATACA	This study
flrA -down-R	TTCAAGTCACGGTTACTGGTTG	This study
Primers for gibson cloning		
pBAD-F	CAGTAGAGAGTTGCGATAAA	This study
pBAD-R	GATGAGAGAAGATTTTCAGC	This study
flrA-pBAD-gibson-F	TTTTTATCGCAACTCTCTACTGATGCAGAGTTTAGCGAAACT	This study
flrA-pBAD-gibson-R	GGCTGAAAATCTTCTCTCATCCTAGCGTTGCATGTTGTATT	This study
Primers for ARMS-PCR		
flrA -outer-F	ATGATGTTCAAACGGTGCAA	This study
flrA -outer-R	GCCAAGCCATTCATGTTTAA	This study

*flrA*213-innerC-F	TTTGGTCATGAAAAAGGTGC	This study
*flrA*213-innerT-R	GTAATCGCTCCGGTAACCA	This study
*flrA*261-innerG-F	GCGCTGTTTTGAACGAGG	This study
*flrA*261-innerT-R	ATGGTGCTGTTGCCTCTCA	This study
**Plasmid**		
pBAD24	Cloning vector with arabinose inducible promoter, Amp^r	[2]
pBAD24::*flrA*	*flrA* gene of WT cloned into MCS site of pBAD24	This study
pBAD24::*flrA* A213V	*flrA* gene containing point mutation affecting amino acid at position 213 cloned into MCS site of pBAD24	This study
pBAD24::*flrA* V261G	*flrA* gene containing point mutation affecting amino acid at position 261 cloned into MCS site of pBAD24	This study
pKD3	FRT-flanked cat gene in oriRγ replicon requiring the *pir* gene product, Addgene plasmid # 45604	[4]
miniTn7(Gm)PrrnB1 –gfpASV	a Gm→,Cm→ PrrnB1 GFP-ASV→ Prr-gfp-ASV cloned into NotI site of pBKminiTn7-ΩGm, *Escherichia coli* AKN139	[5]
miniTn7(Gm)PA1/04/03 –DsRedExpress	A Gm→,Cm→ PA1/04/03 DsRedExpress → PA1/04/03- DsRedExpress (AKN122) cloned into NotI site of pBK-miniTn7-ΩGm, *Escherichia coli* AKN132	[5]
pUX-BF13	oriR6K helper plasmid, mob/oriT, provides Tn7 transposition function in trans, *Escherichia coli* AKN69	[5]
pBR-flp	FLP+, k cI857+, k pR from pCP20 integrated into EcoRV site of pBR322	[6]

Supplementary Table 2. Unique genes mutated in adapted and non-adapted populations sequenced at three different time points

	Locus tag	Gene/Gene function	Number	nsSNP	sSNP	INDEL	Same lineage	Day 3	Day 45	Day 90
Unique to Adapted	VC0760	histidine tRNA ligase	1	1	0	0	-	+	-	-
	VC1180	cysteine/glutathione ABC transporter CydC	1	1	0	0	-	-	-	+
	VC1590	acetolactate synthase AlsS	1	0	1	0	-	+	+	-
	VC1927	C4-dicarboxylate ABC transporter permease	1	1	0	0	-	-	-	+
	VC2021	beta-ketoacyl-ACP reductase	1	1	0	0	-	+	-	-
	VC2137	sigma-54-dependent transcriptional regulator, *flrA*	7	6	0	1	+	-	+	+
	VC2384	hypothetical protein VC2384	1	0	1	0	-	-	-	+
	VC2534	magnesium transporter	1	1	0	0	-	+	-	-
	VC0093	glycerol-3-phosphate 1-O-acyltransferase	1	1	0	0	-	+	-	-
	VC0215	cysteine ligase and decarboxylase CoaBC	1	1	0	0	-	+	-	-
	VC0180	2-dehydropantoate 2-reductase	1	1	0	0	-	+	-	-
	VCA0101	EAL domain-containing protein	1	0	1	0	-	-	-	+
Unique to Non-adapted	VC0534	RNA polymerase sigma factor RpoS	2	0	0	2	-	-	+	-
	VC0893	flagellar motor protein MotB	3	2	0	1	-	-	+	+
	C1H56_06465	resolvase	1	1	0	0	-	-	-	+
	C1H56_07180	ABC transporter permease	1	0	0	0	-	-	+	-
	VC1710	PAS domain S-box protein	1	1	0	0	-	-	+	-
	VC2156	outer membrane protein assembly factor BamC	1	0	1	0	-	-	+	-
	VC2185	redox-regulated ATPase YchF	1	0	1	0	-	-	-	+
	VC2601	Sodium type flagellar protein MotX	1	0	0	1	+	-	+	+
	VCA0171	VWA domain-containing protein	1	1	0	0	-	-	-	+
	VCA0678	periplasmic nitrate reductase subunit alpha	1	1	0	0	-	+	-	-

Supplementary Table 3. Common genes mutated in adapted and non-adapted populations sequenced at three different time points

		Adapted								Non-adapted							
**Locus tag**	**Gene/Gene function**	**Number**	**nsSNP**	**sSNP**	**indel**	**Same lineage**	**Day 3**	**Day 45**	**Day 90**	**Number**	**nsSNP**	**sSNP**	**indel**	**Same lineage**	**Day 3**	**Day 45**	**Day 90**
VC0027	threonine ammonia-lyase, biosynthetic	1	1	0	0	-	+	-	-	2	1	1	0	-	+	+	-
VC0766	exodeoxyribonuclease VII large subunit	1	1	0	0	-	-	+	-	1	1	0	0	+	-	+	+
VC0791	sensor histidine kinase citA	6	5	1	0	+	+	+	+	6	6	0	0	+	+	+	+
VC0905	methionine ABC transporter MetQ	1	1	0	0	-	+	-	+	4	3	1	0	+	+	+	+
VC0913	MexH family multidrug efflux RND transporter	4	4	0	0	+	+	+	+	3	3	0	0	+	+	+	+
VC0995	PTS N-acetylmuramic acid transporter IIBC	1	0	1	0	-	+	+	-	3	1	2	0	+	+	+	+
VC0998	Ploar transmembrane protein	50	23	27	0	+	+	+	+	46	19	27	0	+	+	+	+
VC1015	electron transport complex subunit RsxC	7	0	7	0	+	+	+	+	8	0	8	0	+	+	+	+
VC1162	ATP-dependent Zn protease	2	0	1	1	+	+	+	+	4	2	1	1	-	+	+	+
VC1205	imidazolonepropionase	16	14	2	0	+	+	+	+	16	14	2	0	+	+	+	+
VC1386	molecular chaperone	2	2	0	0	+	+	+	+	3	3	0	0	+	+	+	+
VC1451	multifunctional autoprocessing toxin RtxA	2	2	0	0	-	-	+	+	5	5	0	0	+	+	+	+
VC1455	XRE family transcriptional regulator	1	1	0	0	-	-	+	+	1	1	0	0	-	+	-	-
VC1571	cytochrome ubiquinol oxidase subunit I	2	2	0	0	-	+	+	+	2	2	0	0	+	+	+	+
VC1580	LysR family transcriptional regulator	1	0	1	1	-	+	-	-	1	0	1	0	-	-	-	+
VC1665	ABC transporter permease	1	1	0	0	+	+	+	+	2	2	0	0	+	+	+	+
VC1798	ATPase AAA	1	1	0	0	-	-	+	-	1	1	0	0	-	+	+	+
VC1821	PTS fructose transporter subunit IIC	16	13	3	0	+	+	+	+	16	12	4	0	+	+	+	+
VC2376	glutamate synthase large subunit	4	3	1	0	-	+	+	-	1	1	0	0	-	+	-	-
VC2618	aspartate aminotransferase family protein	1	1	0	0	+	-	+	+	2	2	0	0	-	-	+	+
VC2749	nitrogen regulation protein NR(I)	1	0	1	0	+	-	+	+	1	0	1	0	+	+	+	+
VC0143	hypothetical protein, VC0143	1	1	0	0	-	-	+	+	1	1	0	0	-	+	-	-
VC0171	nickel/dipeptide/oligopeptide ABC transporter	2	2	0	0	-	+	-	-	3	3	0	0	-	-	-	+
VCA0268	methyl-accepting chemotaxis protein	1	1	0	0	-	+	-	-	1	1	0	0	-	+	+	-
VCA0517	1-phosphofructokinase	1	1	0	0	-	+	-	-	1	1	0	0	-	+	-	-

Supplementary Table 4. Unique genes mutated in adapted and non-adapted isolates sequenced at three different time points

	Locus tag	Gene/Gene function	Number	nsSNP	sSNP	INDEL	Day 3	Day 45	Day 90
Unique to Adapted	VC0744	protein translocase subunit SecF	1	1	0	0	-	-	+
	VC0778	Fe(3+)-siderophore ABC transporter permease	1	1	0	0	+	-	-
	VC2137	sigma-54-dependent transcriptional regulator, *flrA*	2	2	0	0	-	+	+
	VCA0283	hypothetical protein	1	0	0	1	-	+	-
Unique to Non-adapted	VC0766	exodeoxyribonuclease VII large subunit	1	1	0	0	+	-	-
	VC0791	sensor histidine kinase	1	1	0	0	-	-	+
	VC0905	methionine ABC transporter MetQ	1	1	0	0	-	-	+
	VC0913	MexH family multidrug efflux RND transporter	1	1	0	0	-	+	+
	VC1451	multifunctional autoprocessing toxin RtxA	1	1	0	0	+	-	-
	VC1798	ATPase AAA	1	1	0	0	+	-	-
	VC2185	redox-regulated ATPase YchF	1	0	1	0	-	-	+
	VC2677	DNA-binding transcriptional regulator CytR	1	0	1	0	+	-	-

Supplementary Table 5. Common genes mutated in adapted and non-adapted isolates sequenced at three different time points

		Adapted							Non-adapted						
**Locus tag**	**Gene/Gene function**	**Number**	**nsSNP**	**sSNP**	**indel**	**Day 3**	**Day 45**	**Day 90**	**Number**	**nsSNP**	**sSNP**	**indel**	**Day 3**	**Day 45**	**Day 90**
VC0998	Polar transmembrane protein	19	9	10	0	+	+	+	12	7	5	0	+	+	+
VC1015	electron transport complex subunit RsxC	2	0	2	0	+	+	+	3	0	3	0	+	-	+
VC1205	imidazolonepropionase	3	3	0	0	-	+	+	4	4	0	0	+	+	-
VC1455	XRE family transcriptional regulator	1	1	0	0	+	-	+	1	1	0	0	+	-	+
VC1821	PTS fructose transporter subunit IIC	6	4	2	0	-	+	+	7	5	2	0	+	+	+
VC2021	beta-ketoacyl-ACP reductase	1	1	0	0	+	+	+	1	1	0	0	-	+	-

Supplementary Data Legends

Supplementary Data 1. Mutations detected in adapted populations.

In the annotation column blue and green coloured represent non-synonymous and synonymous mutation respectively with affected base changes highlighted in red. The percentages represent the frequency of respective mutations found in each replicates.

Supplementary Data 2. Mutations detected in Non-adapted populations.

In the annotation column blue and green coloured represent non-synonymous and synonymous mutation respectively with affected base changes highlighted in red. The percentages represent the frequency of respective mutations found in each replicates.

Supplementary Data 3. Mutations detected in adapted isolates.

In the annotation column blue and green coloured represent non-synonymous and synonymous mutation respectively with affected base changes highlighted in red. The percentages represent the frequency of respective mutations found in each replicates.

Supplementary Data 4. Mutations detected in Non-adapted isolates.

In the annotation column blue and green coloured represent non-synonymous and synonymous mutation respectively with affected base changes highlighted in red. The percentages represent the frequency of respective mutations found in each replicates.

Supplementary references

1. Yildiz FH, Schoolnik GK. Role of rpoS in stress survival and virulence of *Vibrio cholerae*. J Bacteriol. 1998;180:773-84.

2. Espinoza-Vergara G, Noorian P, Silva-Valenzuela CA, Raymond BBA, Allen C, Hoque MM, et al. *Vibrio cholerae* residing in food vacuoles expelled by protozoa are more infectious *in vivo*. Nat Microbiol. 2019;4:2466-74.

3. Cheng T, Kam JY, Johansen MD, Oehlers SH. High content analysis of granuloma histology and neutrophilic inflammation in adult zebrafish infected with *Mycobacterium marinum*. Micron. 2020;129:102782.

4. Datsenko KA, Wanner BL. One-step inactivation of chromosomal genes in *Escherichia coli* K-12 using PCR products. Proc Natl Acad Sci USA. 2000;97:6640.

5. Lambertsen L, Sternberg C, Molin S. Mini-Tn7 transposons for site-specific tagging of bacteria with fluorescent proteins. Environ Microbiol. 2004;6:726-32.

6. Silva ODS, Blokesch M. Genetic manipulation of *Vibrio cholerae* by combining natural transformation with FLP recombination. Plasmid. 2010;64:186-95.
